# Saudi expert consensus on acquired hemophilia A diagnosis and management

**DOI:** 10.1016/j.jtumed.2024.04.006

**Published:** 2024-04-27

**Authors:** Abdulkareem M. Almomen, Hazza A. Alzahrani, Hussein H. AlSaeed, Zohair AlAseri, Ahmed F. Mady, Tarek Owaidah

**Affiliations:** a Department of Pathology, College of Medicine, King Saud University, Riyadh, KSA; b Adult Hematology/HSCT, Oncology Centre, King Faisal Specialist Hospital and Research Center, Riyadh, KSA; c Department of Hematology, Qatif Central Hospital, Qatif Health Network, East Cluster Ministry of Health, KSA; d Departments of Emergency Medicine and Critical Care, College of Medicine, King Saud University, Riyadh, KSA; e ICU, King Saud Medical City, Riyadh, KSA; f Department of Anesthesiology and ICU, Tanta University Hospital, Egypt; g Department of Pathology and Laboratory Medicine, King Faisal Specialist Hospital and Research Centre, Riyadh, KSA; h Alfaisal University, Riyadh, KSA; i Department of Clinical Sciences, College of Medicine and Riyadh Hospital, Dar Al Uloom University, Riyadh, KSA; j Therapeutic Deputyship, Ministry of Health, Riyadh, KSA

**Keywords:** مكتسب, هيموفيليا, إجماع, السعودية, خبير, Acquired, Consensus, Expert, Hemophilia, Saudi

## Abstract

**Objectives:**

Acquired hemophilia affects approximately one in 1 million people. Timely diagnosis is key to appropriate disease management and the prevention of life-threatening complications. Patients with this condition may initially be seen by inexperienced physicians and remain underdiagnosed for several years. This consensus statement is aimed at providing guidelines for all practitioners in the Kingdom of Saudi Arabia (KSA) to diagnose and manage acquired hemophilia A.

**Methods:**

This consensus statement reflects the opinions drafted by a group of hematology specialists, who used an explicit systematic process to identify areas of agreement and disagreement.

**Results:**

This consensus statement provides a guide for all practitioners in the KSA regarding the diagnosis of clinical presentation, relevance, characteristics of bleeding symptoms, and case management; it additionally provides guidance for non-specialists. All management aspects, including diagnosis and treatment modalities, are discussed.

**Conclusions:**

Patients with acquired hemophilia may initially be seen by physicians who lack appropriate expertise in diagnosing and managing this condition. This consensus statement from the premier experts on the disease in the KSA provides details for diagnosing and managing acquired hemophilia.

## الملخص

**أهداف البحث:**

يصاب حوالي شخص واحد من كل مليون شخص بالهيموفيليا المكتسبة. يعد التشخيص في الوقت المناسب أمرا بالغ الأهمية لإدارة المرض بطريقة مناسبة والوقاية من المضاعفات التي تهدد الحياة. قد يلجأ هؤلاء المرضى في البداية إلى أطباء غير متخصصين وقد يبقون غير مشخصين لسنوات عدة. يهدف هذا الإجماع إلى توفير إرشادات لجميع الممارسين في المملكة العربية السعودية لتشخيص وإدارة الهيموفيليا المكتسبة.

**طرق البحث:**

تعكس هذه البيانات الإجماعية الآراء التي تم صياغتها بواسطة مجموعة من أخصائيي أمراض الدم باستخدام عملية منهجية صريحة لتحديد مجالات الاتفاق والاختلاف.

**النتائج:**

يمكن استخدام هذا البيان الإجماعي كدليل لجميع الممارسين في المملكة العربية السعودية فيما يتعلق بتشخيص المظاهر السريرية، والعلاقة، وخصائص أعراض النزيف، وإدارة هذه الحالات، مع توجيه خاص للأخصائيين غير المتخصصين. سوف يناقش الإجماع جميع جوانب الإدارة، من التشخيص إلى أساليب العلاج.

**الاستنتاجات:**

قد يلجأ مرضى الهيموفيليا المكتسبة في البداية إلى أطباء يفتقرون إلى الخبرة المناسبة في تشخيص وإدارة هؤلاء المرضى. يوفر هذا البيان الإجماعي وثيقة مفصلة لتشخيص وإدارة الهيموفيليا المكتسبة، بتوجيه من أفضل الخبراء في المرض في المملكة العربية السعودية.

## Introduction

Acquired hemophilia A (AHA) is an atypical bleeding illness resulting in the generation of autoantibodies to the distinct epitopes of factor VIII in patients with no history of bleeding disorder.^1^ These autoantibodies neutralize FVIII coagulant activity (FVIII:C). Antibodies that bind coagulation components and either decrease or expedite their clearance from the body are known as acquired coagulation inhibitors. However, the inhibitors of neutralizing AHAs differ from those of the alloantibodies directed against FVIII in patients with congenital hemophilia A (HA). Alloantibodies in patients with HA develop after repeated exposure to recombinant or plasma-derived plasma FVIII products that are administered as replacement therapy.^2^ In AHA, the loss of immune tolerance to various genetic and environmental factors can lead to the production of autoantibodies. The prevalence of various comorbid conditions is relatively higher in American patients with AHA than the general population.^3^ According to our observations, we see both autoimmune and cancer associated mainly with lymphoproliferative disorders.

Acquired hemophilia B is caused by autoantibodies directed against factor IX; few cases have been described to date.^2^ Because of the extreme rarity of the disorder, there is lack of experience in Hemophilia B treatment. However, appropriate investigations must be made for its diagnosis in the case of normal FVIII C values like in acquired von Willebrand syndrome. The hereditary form of this bleeding disorder yields test findings and clinical signs equivalent to those associated with the inherited form.^4^

## Materials and Methods

Because our consensus statement does not involve patient data, an institutional review board waiver was granted.

### Database searches

The literature search was performed in indexed online databases MEDLINE/PubMed with the key search terms “acquired h(a)emophilia,” “h(a)emophilia with inhibitors,” “acquired factor VIII inhibitors,” and “acquired inhibitors."

### Data extraction

The full text of relevant abstracts was retrieved, and literature from the authors' libraries was added as a supplement. A total of 150 articles were retrieved and assigned to different authors. Two authors independently reviewed the full text of the studies included in the analysis and extracted study data to determine their relevance to the search aim. These steps were performed to ensure the accuracy of the results.

Abbreviations: Kingdom of Saudi Arabia (KSA), acquired hemophilia A (AHA), FVIII coagulant activity (FVIII:C), hemophilia A (HA), prothrombin time (PT), activated partial thromboplastin time (aPTT), factor VIII (FVIII), lupus anticoagulant (LA), Bethesda units (BU), VIII deficient plasma (VIII-def), buffered normal plasma pool (B-NPP), enzyme-linked immunosorbent assay (ELISA), recombinant activated factor VII (rFVIIa), activated prothrombin complex concentrates (APCC).

## Clinical features

The estimated incidence of AHA is approximately six cases per 1 million people per year.^5^ The incidence rate increases with age. More than 80% of patients are ≥65 years of age, and very few cases are reported in children.^2^ According to cohort-based studies, the median age at diagnosis ranges from 74 to 78 years. Age distribution patterns for FVIII autoantibodies are characteristically biphasic. A small peak is observed between 20 and 30 years of age, owing to postpartum inhibitors, and a considerable increase is observed in the older population. The incidence rate of AHA is similar between men and women, except in the 20–40 year age group, in which more cases are found in women than men, because of pregnancy-associated postpartum bleeding.^6^

Malignancy, pregnancy, and autoimmune illnesses (such as rheumatoid arthritis and systemic lupus erythematosus) are recognized risk factors for AHA. In contrast, nearly half of all cases have unknown causes and are categorized as idiopathic.^2^

The bleeding pattern in AHA is distinct from that in congenital hemophilia. Epistaxis, gastrointestinal and urological bleeding, retroperitoneal hematomas, and postpartum bleeding are common types of bleeding that can occur in the body. In contrast, hemarthrosis, a common feature of congenital FVIII deficiency, has been reported in only a small number of patients with AHA. The bleeding manifestations of AHAs vary, ranging from life threatening forms to mild or no bleeding. Although rare, AHA is therefore considered a serious bleeding disorder with a high fatality rate. Severe threatening bleeding requiring hemostatic support or transfusion treatments can occur in an estimated 70–90% of patients and is fatal in approximately 5–10% of cases. The clinical effects of AHAs are complicated by diagnostic delays and inadequate treatment.^6^

The rarity of AHA cases has contributed to the lack of sufficient supporting data to develop proper diagnosis and management plans. Treatment decisions are often devised according to the clinical expertise of the treating physician, and patients are referred to expert centers for the best possible disease management.^7^ Early detection and physician awareness are imperative for improving disease prognosis. Most AHA cases have been reported in pregnant women in the Kingdom of Saudi Arabia (KSA). Here, we provide an updated set of guidelines and recommendations based on recently available higher level evidence and the experience of a panel of experts from the KSA, to improve evidence-based clinical practice in AHA treatment.

### Recommendation

We recommend that clinicians evaluate patients with suspected or confirmed AHA, with or without bleeding, and refer them as soon as possible to a hemophilia center with expertise in inhibitor management.

## Diagnosis

Patients’ clinical presentation, which signals AHA and is followed by appropriate laboratory knowledge to commence a good diagnostic inquiry, are the primary factors considered in the initial diagnosis. Because AHA can refer to acquired factors, the initial recognition is based on changes observed in routine clotting tests. When bleeding with clinical suspicion of factor deficiency is observed, as part of the preliminary investigation, a blood sample must be sent to a laboratory to determine the activated partial thromboplastin time (aPTT) and prothrombin time (PT).

Because the most common acquired factor deficiency is FVIII, which usually results in prolonged aPTT, the initial workup should include the following:

1 Pharmacological anamnesis, particularly for antithrombotic therapies (heparins, direct oral anticoagulants, and vitamin K antagonists)
2 Collection of blood samples according to standard guidelines
3 Adequate quality control
4 Mixing study
5 Exclusion of the presence of heparin in the sample
6 Exclusion of the presence of lupus anticoagulant (LA)
7 Factor assays, starting with the most common, FVIII
8 Identification of the inhibitor (Bethesda assay)
9 Interpretation of the results and clinical findings

The laboratory diagnosis is described in detail in APPENDIX I.

### Recommendation

We strongly suggest that a diagnosis of acquired hemophilia be considered in any patient who has just started bleeding or has an unexplained extended aPTT but normal PT. We recommend using an algorithm for differential diagnosis of isolated prolonged aPTT.

## Management

Management of AHAs has two primary objectives. The first is to achieve homeostasis, and the second is to eradicate inhibitors. The priority of AHA management is to treat cases of life-threatening bleeding and decrease the risk of future bleeding. Clinicians worldwide use various modalities for treating bleeding and preventing future bleeding, mainly through inhibitor eradication.

### Treatment of bleeding episodes

As discussed earlier, treatment of bleeding episodes is aimed at managing the bleeding.

#### Bypassing agents (APCC and rFVIIa)

For acute severe bleeding, bypassing agents are considered the first treatment option.^8^ However, prophylactic use of these agents has also been reported in patients with AHA, to prevent the risk of recurrent bleeding in vulnerable patients, particularly before the use of more invasive procedures.^9^ Two bypassing agents are currently used to restore homeostasis:

• Recombinant activated factor VII (rFVIIa), available as Novoseven™, is usually administered at 90 μg/kg every 2–3 h.
• Activated prothrombin complex concentrates (APCC), available as FEIBA™, a plasma-derived concentrate, is administered every 8–12 h at doses of 75 IU/kg, not exceeding 200 IU/kg/day.^10^

The choice between rFVIIa and APCC depends on patient characteristics, including previous treatment response and injection availability, required frequency, and physician experience.^9^ Treatment efficiency is assessed on the basis of hemoglobin levels, and clinical examination combined with the results of repeat imaging.^9^

#### Factor VIII concentrate

Human FVIII concentrate is used for treating patients with AHA with low inhibitor titers (<5 Bethesda units [BU]) or when bypassing agents are not immediately available. Effective doses are calculated with various formulas.^11^ Treatment success is assessed according to FVIII plasma levels. However, bleeding risk cannot be predicted solely on the basis of inhibitor titers or residual FVIII levels.^11^

#### Recombinant porcine factor VIII

In Canada, Europe, and the US, recombinant porcine factor VIII has recently been approved for treating acute bleeding episodes in patients with AHA. The approved dose is 200 U/kg, and subsequent doses are necessary to maintain FVIII trough levels greater than 50%. The molecule is well-tolerated; the most common adverse event is the formation of antibodies to r-pFVIII. Cross-reacting inhibitors are relatively more common in individuals with anti-hFVIII inhibitor titers exceeding 100 BU/mL, according to two recent independent investigations. After approval of this drug, several studies and case series have described cohorts of individuals treated with r-pFVIII for AHA, all of which have confirmed the effectiveness and safety of susoctocog alfa in treating bouts of severe bleeding (47, 48, 49).

#### Desmopressin (DDAVP)

This therapeutic agent is recommended for non-life-threatening bleeding as intravenous 0.3 to 0.4 mcg/kg once. With this therapy, similarly to FVIII concentrates, the main concerns are associated with potential efficiency. Another concern is the increased risk of tachyphylaxis with subsequent doses. Secondary hyponatremia and water retention must be monitored in older patients.^2^

#### Emicizumab

Emicizumab (Hemlibra®, Roche, USA) is a bispecific humanized monoclonal antibody that mimics the function of absent FVIII in individuals with HA by facilitating the interaction between activated factor IX and factor X.^12^ Emicizumab restores FVIII function to 10%–20% of the levels occurring naturally in the body, thus resulting in a shift from severe bleeding to mild symptoms.^13^^,^^14^ Emicizumab has shown efficacy in decreasing bleeding in adult and adolescent patients with HA, regardless of the presence of inhibitors.^15^ Moreover, it can be administered via the subcutaneous route and does not require venous access. Emicizumab is commonly used as a preventive therapy in several countries.^16^^,^^17^

#### Tranexamic acid

This agent is used in combination with other hemostatic agents in AHA treatment.^9^

### Eradication of inhibitors

The second objective of AHA management is inhibitor eradication, which aids in long-term disease management.

#### Immunosuppression

Immunosuppression should be initiated immediately after diagnosis of AHA. Corticosteroids alone or in combination with cyclophosphamide can be prescribed as a first-line treatment.^18^ However, immunosuppressive agents carry a heightened risk of infectious diseases; therefore, patients must be monitored closely, and this treatment must be used cautiously in frail patients.^18^

The most common steroid medication, prednisone, is administered in the range of 1–2 mg per kilogram per day for approximately 4–6 weeks.^9^ Cyclophosphamide is orally administered at 1–2 mg/kg/day for 6 weeks. The response is determined on the basis of decreased inhibitor titer or increased FVIII levels after 3–5 weeks of treatment.^9^ Patients diagnosed with AHAs have also been treated with rituximab alone or in conjunction with other medications.^19^

#### Immune tolerance induction

The immune tolerance induction efficacy in AHA is supported by little evidence, and the excessive expense of this treatment limits its application. Human FVIII can induce immunological tolerance and has been used with moderate success in patients with AHA. However, worldwide guidelines discourage the use of immune tolerance induction in AHA.

#### Rituximab

When initial attempts to eliminate the inhibitor are unsuccessful, rituximab may be considered as a potential substitute.^20^ Rituximab is often efficacious in eradicating factor VIII inhibitors after initial therapeutic attempts have failed, according to an extensive body of scientific evidence. Rituximab, a chimeric monoclonal antibody, targets CD20, a transmembrane protein that is ubiquitous on B cells but is absent from mature plasma cells. B cells are depleted from fluidborne and lymphoid tissue by this monoclonal antibody.^21^

### Follow-up

Patients with AHA must be followed up for disease relapse. An estimated 10–20% of patients with AHA experience relapse. FVIII and aPTT levels should be monitored every month during the first 6 months, every 2–3 months during the following 6 months, and every 6 months thereafter.^22^

## Non-hematologist perspectives

The high morbidity and mortality among patients with AH are attributable to several factors, including patient age; underlying diseases; and adverse events associated with treatment, such as infections or sepsis associated with immunosuppressive therapy,^23^ excessive blood loss, and serial delays in diagnosis and appropriate management. Patients might initially be seen by clinicians without prior disease experience. Consequently, greater awareness is critical among healthcare personnel who are likely to come into contact with patients with AH. In addition, we believe that the optimal treatment for AH requires active cooperation among non-hematologist physicians, pharmacists, and laboratory personnel, in addition to hematologists, who specialize in blood disorders.

In a survey of 1104 non-hematologist healthcare professionals across 10 Arabian Gulf tertiary care hospitals, 42% were unaware of AH, while 45% did not consider mixing tests for isolated prolonged aPTT, and nearly half did not consider bypassing agents in bleeding AH. Most non-hematologist clinicians, laboratory staff, and pharmacists agree that the primary impediment to appropriate therapy for AH is a lack of understanding regarding the condition and its complications. Only 4.2% of the cohort did not believe that despite raising awareness, non-hematologist healthcare professionals could impart improved emergency disease management for such a fatal disorder.^24^

AHA is a relatively less prevalent bleeding disorder that occurs unexpectedly. Patients with AHA are often initially seen by physicians in different specialties. Therefore, a simple diagnostic algorithm is needed to help physicians lacking expertise in adequately diagnosing patients with AHA (Figure 2).^8^ Ten challenges for non-hematologists are listed in Table 1, and the differential diagnosis of coagulopathy in the ICU is shown in Table 2.

**Figure 1:**
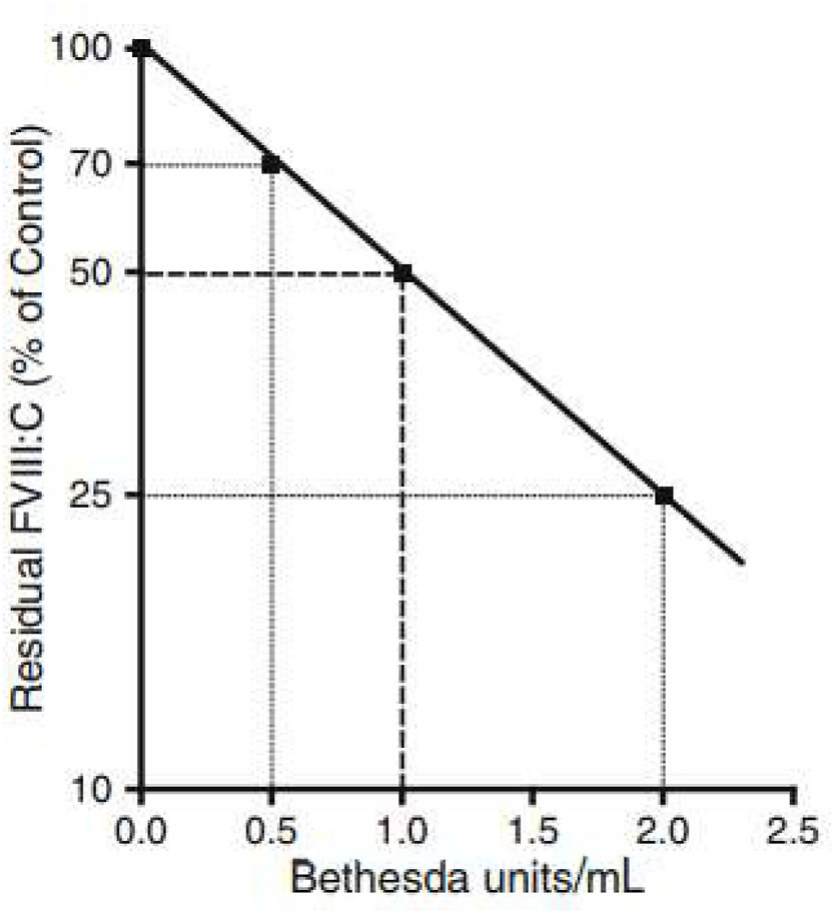
Inhibitor graph correlating the percentage residual FVIII:C to Bethesda units/mL in test plasma.

**Figure 2:**
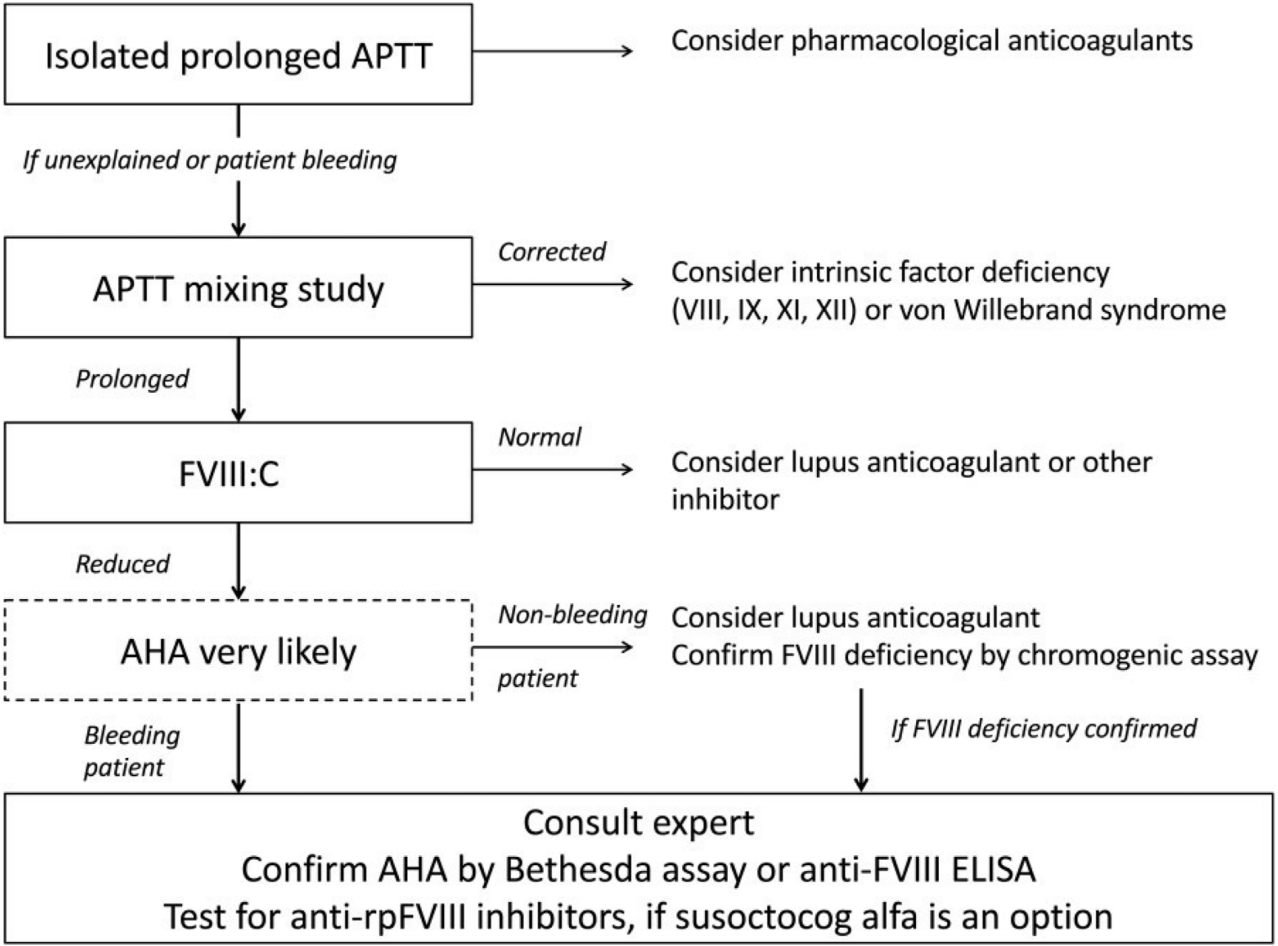
Pathway for diagnosing and managing acquired hemophilia A. If a factor VIII (FVIII) activity assay is immediately available, the activated partial thromboplastin time (aPTT) mixing study might be unnecessary. Another critical point of consideration is that the presence of lupus anticoagulant does not necessarily exclude the diagnosis of acquired hemophilia A. AHA: acquired hemophilia A; FVIII:C: factor VIII activity; rpFVIII: recombinant porcine factor VIII; ELISA: enzyme-linked immunosorbent assay.

**Table 1:** Challenges for non-hematologists.

1. Severity of clinical presentation and rarity of the disease
2. Tendency of patients to present to non-specialist physicians for AHA
3. Delays between bleeding onset and diagnosis
4. Requirements for specialist clinical and laboratory expertise and facilities
5. Frequent confusion with other life-threatening conditions (e.g., DIC)
6. Absence of high-level evidence to support management recommendations
7. Unavailability and/or restriction of hemostatic medications
8. Risk of various adverse effects with use of immunosuppressive agents, predominantly in older age groups
9. Elevated risk of thrombotic complications due to cardiovascular comorbidities associated with advanced age in patients with AHA during hemostatic therapy
10. Postponement of procedures until inhibitor eradication has been achieved, whenever possible

**Table 2:**
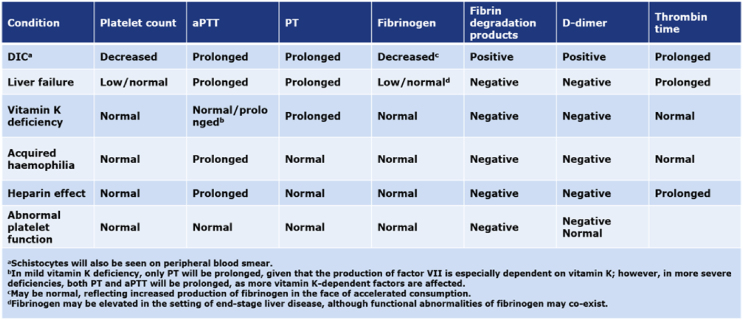
Most common coagulation disorders in intensive care units.

Approximately 10% of patients with AHAs do not present with bleeding. Prolonged aPTT should be considered before invasive procedures are performed on these patients.^8^ In patients with AHA, significant blood loss may occur, even with minor invasive procedures^24^; hence, all these procedures should be performed with great caution, and if possible, surgery should be delayed until inhibitor eradication has been achieved. Bypassing agents are recommended for biopsies, central venous access, or invasive procedures.^8^

According to the AHA Working Group of the German, Austrian, and Swiss Thrombosis and Haemostasis Society and surveillance registries, cardiovascular events, such as myocardial infarction, stroke, and thrombosis, are reported as the cause of death in 6–7% of patients with AHA.^25^ Therefore, thromboprophylaxis can be recommended in non-bleeding patients with normal FVIII:C levels, according to the 2018 American Society of Hematology guidelines.^26^ If patients have a history of disorders that require treatment with antiplatelet drugs or oral anticoagulants, physicians must wait for FVIII levels to return to normal before prescribing these agents.^26^

### Recommendation

AHA is a relatively less prevalent bleeding disorder, and patients with this disorder are frequently initially seen by physicians in different specialties. Non-hematologist physicians, pharmacists, and laboratory personnel must collaborate closely with hematologists to achieve the best potential treatment outcomes for patients with AH. Hematologists are the only medical specialists capable of diagnosing and treating AH. To more effectively treat the condition, increasing the knowledge among healthcare professionals who are likely to interact with patients with AHA crucial. A simple diagnostic algorithm can assist physicians lacking adequate expertise in adequately diagnosing patients with AHA (Figure 2).

## Disclaimer

Alyaan Consult Agency provided medical writing, statistical analysis, and editorial assistance. Novo Nordisk provided financial support for the research. The results and interpretations of this study are the sole responsibility of the authors, who are willing to accept full responsibility for them. Novo Nordisk did not alter the content of this publication, nor was the company involved in the design of the study, data collection, data interpretation, or data review. Moreover, the company did not participate in the data analysis.

## Funding

This research did not receive any specific grant from funding agencies in the public, commercial, or not-for-profit sectors.

## Conflict of interest

The authors declare that they have no known competing financial interests or personal relationships that could have appeared to influence the work reported in this paper.

## Ethical approval

There are no ethical issues.

## Consent for publication

All authors consent for publication.

## Author contributions

TO and AA designed and developed the study. HAA and HHA are responsible for the content and authenticity, and oversaw data collection and entry. ZA and AM performed a final review of the data and analysis. All authors are responsible for the study team's direction and the project plan's facilitation. All authors significantly contributed to the work reported, in the conception, study design, execution, acquisition of data, analysis, and/or interpretation, and took part in drafting, revising, or giving final approval of the version to be published in the journal to which the article has been submitted. All authors have critically reviewed and approved the final draft and are responsible for the content and similarity index of the manuscript.

## Data sharing

Furnished upon reasonable request to the corresponding author.

## Availability of data and materials

Furnished upon request.

## Disclosure

No part of the article was presented in conference proceedings.

## Appendix

1. Sample collection

The duration of time between sample collection and analysis can affect the outcomes of clotting-based assays.^27^ Whereas storage at temperatures of 2–8 °C activates many proteolytic systems, storage at temperatures above 25 °C decreases FVIII activity over time.^28^ If blood samples are held at temperatures of 2–8 °C before processing, FVIII and VWF can be lost, thus potentially leading to misdiagnosis of healthy patients with von Willebrand disease. Therefore, the recommended storage time is as many as 3.5 h at room temperature. However, for longer storage at 4 °C, samples must be pre-warmed before testing.

2. Quality control

Qualified laboratory personnel must collect venous blood specimens in plastic tubes with non-activating surfaces for coagulation testing. The tubes should contain 3.2% buffered trisodium citrate as the anticoagulant. A 9:1 proportion of blood to anticoagulant must be maintained.^29^ Every laboratory must define specific criteria for rejecting specimens unsuitable for coagulation testing. These criteria must include rejection of unlabeled or mislabeled specimens, specimens that do not contain blood and anticoagulants in appropriate proportions, specimens collected in sample tubes with the incorrect anticoagulant, clotted specimens, grossly hemolyzed specimens, specimens that do not arrive within the appropriate timeframe for analysis, and specimens that have been stored in a refrigerator before analysis.^27^

3. Mixing study

The aPTT assay is typically considered a reliable screening test for detecting factor VIII (FVIII) inhibitors, because aPTT is usually prolonged when FVIII activity falls to 45% or less of the mean normal value. In addition, mixing normal plasma with patient plasma will not normalise the aPTT and reduce FVIII activity.^30^ Therefore, the diagnosis must consider the isolated extended aPTT associated with partial correction estimated after a 1:1 mixing trial, both immediately and after a 2-h incubation.^18^

a. Exclusion of the presence of heparin in a sample

Heparinoids, heparins, and direct oral anticoagulants can mimic circulating inhibitors and interfere with inhibitor test results, thus leading to false diagnoses. The exclusion of their presence from the sample is a prerequisite.

b. Lupus anticoagulant

LA can occasionally coexist with an FVIII inhibitor, and its presence must be considered before the final diagnosis. In such cases, the diluted Russel viper venom time test can be used to detect LA. However, the presence of LA does not exclude AHA.^31^

4. Factor assay

A single-stage FVIII:C test or a chromogenic assay can be used. The single-stage clot-based assay is primarily an activated PTT-based test frequently used in clinical laboratories. FVIII-deficient plasma is mixed with the patient sample, and the mixture is pre-incubated with aPTT reagent with a contact activator and phospholipid. Adding calcium chloride to the mixture accelerates the formation of a fibrin clot, the endpoint for aPTT measurement. In calculating blood clotting time, the patient's sample FVIII concentration is largely believed to be the rate-limiting component. The assay result is compared with a standard curve, and the patient's FVIII activity is interpolated.^32^

a. Inhibitor identification

Diagnosis is verified by demonstration of a low FVIII:C level and the presence of an FVIII inhibitor with the Nijmegen-modified Bethesda assay. The diagnosis is confirmed when a low FVIII:C level has been demonstrated. The results of this assay are reported in Bethesda units (BU).^9^ Inhibitors are dependent on time and temperature. Incubation for 2 h at 37 °C is required before detection. AHA autoantibodies are primarily type 2 inhibitors in some cases. Therefore, they exhibit complex inhibition kinetics and cannot completely neutralize FVIII:C. HA type 1 inhibitors, in contrast, follow second-order kinetics and thus completely inactivate FVIII:C. The sensitivity of inhibitor detection can be increased by heat treatment of the sample before the assay (58 °C for 90 min) and by eliminating residual FVIII.^9^

A mix of equal volumes (300 μL) for all incubations can provide sufficient final volumes for analysis with most automated analyzers. For preparation of control tubes, an equal volume of VIII-deficient plasma (VIII-def) is mixed with a buffered normal plasma pool (B-NPP). Full strength and ½ dilution tests are performed for diagnosis. The mixtures are incubated for 2 h at 37 °C, and the residual FVIII:C is measured in control and test mixtures.

5. Result interpretation

The ratio of residual factor VIII is then calculated:

$$\text{Residual}\_\text{FVIII}\_\text{rati}=\left(\frac{\text{FVIII}:C\_\text{patient}\_\text{mixture}}{\text{FVIII}:C\_\text{control}\_\text{mixture}}\right)$$

The ratio is converted to a percentage. If the ratio results range from 25% to 75% remaining FVIII:C, the sample's BU/mL is estimated with the theoretical inhibitor graph. The percentage residual activity is interpolated against BUs (Figure 1). Incubation mixtures with >75% residual FVIII:C do not contain any detectable inhibitor (<0.5 BU/mL). Analysis of samples with elevated inhibitor levels and <25% residual FVIII:C must be repeated after appropriate dilution to the 25–75% range.

The novel Bethesda method was developed primarily for standardizing the measurement of inhibitors during FVIII neutralization assays. One BU is thus defined as an inhibitor that yields 50% residual FVIII:C activity in a test mixture. In a more recent Nijmegen variation, the protein concentration and pH of the test mixture were more standardized; thus, FVIII:C in the test mixture was less inclined toward artifactual deterioration, and specificity was increased.^33^

6. Enzyme-linked immunosorbent assay

Additionally, an anti-FVIII enzyme-linked immunosorbent assay, performed primarily after thermal treatment of samples, has been proposed.^34^

## References

[1] Sholzberg M. (2018).

[2] Mazzucconi M.G., Baldacci E., Ferretti A., Santoro C. (2020). Acquired haemophilia A: an intriguing disease. Mediterranean J Hematol Infect Dis.

[3] Soucie J.M., Le B., Dupervil B., Poston J.N. (2022). Prevalence of comorbid conditions among older males with haemophilia receiving care in haemophilia treatment centers in the United States. Haemophilia.

[4] Itzhar-Baikian N., Boisseau P., Joly B., Veyradier A. (2019). Updated overview on von Willebrand disease: focus on the interest of genotyping. Expert Rev Hematol.

[5] Tiede A., Wahler S. (2021). The rising incidence of acquired haemophilia A in Germany. Haemophilia.

[6] Franchini M., Vaglio S., Marano G., Mengoli C., Gentili S., Pupella S. (2017). Acquired hemophilia A: a review of recent data and new therapeutic options. Hematology.

[7] Tiede A., Collins P., Knoebl P., Teitel J., Kessler C., Shima M. (2020). International recommendations on the diagnosis and treatment of acquired hemophilia A. Haematologica.

[8] Tiede A., Collins P., Knoebl P., Teitel J., Kessler C., Shima M. (2020). International recommendations on the diagnosis and treatment of acquired hemophilia A. Haematologica.

[9] Charlebois J., Rivard G.-É., St-Louis J. (2018). Management of acquired hemophilia A: review of current evidence. Transfus Apher Sci.

[10] Poon M.-C. (2021). The use of recombinant activated factor VII in patients with Glanzmann's thrombasthenia. Thromb Haemostasis.

[11] Amano K., Seita I., Higasa S., Sawada A., Kuwahara M., Shima M. (2017). Treatment of acute bleeding in acquired haemophilia A with recombinant activated factor VII: analysis of 10-year Japanese postmarketing surveillance data. Haemophilia.

[12] Kitazawa T., Igawa T., Sampei Z., Muto A., Kojima T., Soeda T. (2012). A bispecific antibody to factors IXa and X restores factor VIII hemostatic activity in a hemophilia A model. Nat Med.

[13] Donners A.A., Rademaker C.M., Bevers L.A., Huitema A.D., Schutgens R.E., Egberts T.C. (2021). Pharmacokinetics and associated efficacy of emicizumab in humans: a systematic review. Clin Pharmacokinet.

[14] Kizilocak H., Marquez-Casas E., Malvar J., Carmona R., Young G. (2021). Determining the approximate factor VIII level of patients with severe haemophilia A on emicizumab using in vivo global haemostasis assays. Haemophilia.

[15] Young G., Liesner R., Chang T., Sidonio Jr R., Oldenburg J., Jiménez-Yuste V. (2019). A multicenter, open-label phase 3 study of emicizumab prophylaxis in children with hemophilia A with inhibitors. Blood, The Journal of the American Society of Hematology.

[16] Mahlangu J., Iorio A., Kenet G. (2022). Emicizumab state-of-the-art update. Haemophilia.

[17] Belletrutti M., Bhatt M., Samji N. (2023). Management of children with hemophilia A on emicizumab who need surgery. Front Pediatr.

[18] Kruse-Jarres R., Kempton C.L., Baudo F., Collins P.W., Knoebl P., Leissinger C.A. (2017). Acquired hemophilia A: updated review of evidence and treatment guidance. Am J Hematol.

[19] Remmington T., Smith S. (2021). Rituximab for eradicating inhibitors in people with acquired haemophilia A. Cochrane Database Syst Rev.

[20] Jiang L., Liu Y., Zhang L., Santoro C., Rodriguez A. (2020). Rituximab for treating inhibitors in people with inherited severe hemophilia. Cochrane Database Syst Rev.

[21] Franchini M. (2007). Rituximab in the treatment of adult acquired hemophilia A: a systematic review. Crit Rev Oncol Hematol.

[22] Arruda V.R., Lillicrap D., Herzog R.W. (2022). Immune complications and their management in inherited and acquired bleeding disorders. Blood J Am Soc Hematol.

[23] Mingot-Castellano M.E., Rodríguez-Martorell F.J., Nuñez-Vázquez R.J., Marco P. (2022). Acquired haemophilia A: a review of what we know. Hematol Res Rev.

[24] Mady A.F., Huwait B., Rana M.A., Ramadan O.E., Al-Harthy A., Alatribi W.T. (2018). Awareness and perspectives on nom haematologist in the management of acquired haemophilia in Arab gulf countries. Esculapio April–June.

[25] Franchini M., Schiavulli M., Liumbruno G.M. (2021). Hemostatic therapy as a management strategy for acquired hemophilia: what does the future hold?. Expert Rev Hematol.

[26] Schünemann H.J., Cushman M., Burnett A.E., Kahn S.R., Beyer-Westendorf J., Spencer F.A. (2018). American Society of Hematology 2018 guidelines for management of venous thromboembolism: prophylaxis for hospitalized and nonhospitalized medical patients. Blood Adv.

[27] Bronić A., Coen Herak D., Margetić S., Milić M. (2019). Croatian Society of Medical Biochemistry and Laboratory Medicine: national recommendations for blood collection, processing, performance and reporting of results for coagulation screening assays prothrombin time, activated partial thromboplastin time, thrombin time, fibrinogen and D-dimer. Biochem Med (Zagreb).

[28] Toulon P., Metge S., Hangard M., Zwahlen S., Piaulenne S., Besson V. (2017). Impact of different storage times at room temperature of unspun citrated blood samples on routine coagulation tests results. Results of a bicenter study and review of the literature. Int J Lab Hematol..

[29] Lima-Oliveira G., Brennan-Bourdon L., Varela B., Arredondo M., Aranda E., Flores S. (2021). Clot activators and anticoagulant additives for blood collection. A critical review on behalf of COLABIOCLI WG-PRE-LATAM. Crit Rev Clin Lab Sci.

[30] Ovanesov M.V., Jackson J.W., Golding B., Lee T.K. (2021). Considerations on activity assay discrepancies in factor VIII and factor IX products. J Thromb Haemostasis.

[31] Moore G., Peyrafitte M., Dunois C., Amiral J. (2018). Newly developed dilute Russell's viper venom reagents for lupus anticoagulant detection with improved specificity. Lupus.

[32] Novembrino C., Boscolo Anzoletti M., Mancuso M.E., Shinohara S., Peyvandi F. (2019). Evaluation of an automated chromogenic assay for Factor VIII clotting activity measurement in patients affected by haemophilia A. Haemophilia.

[33] Kaneda M., Kawasaki R., Matsumoto N., Abe H., Tashiro Y., Inokuchi Y. (2021). Detailed analysis of anti-emicizumab antibody decreasing drug efficacy, using plasma samples from a patient with hemophilia A. J Thromb Haemostasis.

[34] Chansavang A., Philippe A., Bozinovic I., Ben Hadj Ali K., Smadja D., Helley D. (2022). Usefulness of anti-factor VIII IgG ELISA in acquired hemophilia A follow-up. Ann Hematol.

